# Personal Experiences in Empyema of the Maxillary Sinus

**Published:** 1893-03

**Authors:** H. Gradle

**Affiliations:** Chicago, Ill.


### Personal Experiences in Empyema of the Maxillary Sinus.

BY H. GRADLE, M.D.,
OF CHICAGO, ILL.

Read in the Section of Oral and Dental Surgery, at the Forty-third Annual Meeting of the American Medical Association, held at Detroit, Mich., June, 1892.

Suppuration of the antrum is a subject so much discussed of late that it would be unprofitable to present to you anything but personal experiences on mooted points. The diagnosis of this trouble is easy only in cases with pronounced symptoms, but in very many it is quite difficult to be certain of it. In cases coming under your observation as dentists the dental history is often a definite guide in the diagnosis. In empyema, however, of nasal origin, considerable diagnostic difficulty may be experienced.

The only constant symptom is discharge from the nose. If this be one-sided there can be scarcely any doubt that it comes from one of the accessory cavities, but not necessarily from the antrum of Highmore. When disease involves both sides, however, this criterion fails. A fetid odor strongly suggests retention of pus in a sinus. The pus is probably always fetid if of dental origin, but not necessarily if the antrum has been infected from the nose. The quantity of the discharge varies in different instances and is no criterion. If the pus can be seen issuing from underneath the middle turbinated bone it must come from either the frontal or the maxillary sinus.

Pain in and around the cheek is common but not constant in acute cases, but infrequent in chronic instances. Tenderness over the inflamed antrum I have never seen.

I have not been able to rely for diagnostic purposes upon transluminatiun of the bone by means of an electric lamp placed in the mouth. In order to use this method the room must be very dark or an opaqne hood must be put over the heads of surgeon and patient. An American lamp which I used at first I found too weak, but I find that I cannot rely either upon an eight-candle power furnished by Hirschman, of Berlin. The transparency varies with the configuration of the maxilla, and pus, unless copious and thick, does not impede the light much more than bone tissue.

An absolute diagnosis can only be made by exploration. This is a very innocent procedure when made from the alveolar process. If the teeth are sound, however, and there is no room between them. a puncture can be made through the canine fossa. It is well to incise the mucous membrane where it is still firmly attached to the alveolar process and to separate it upward from the bone. For if the loose tissue under the cheek is irritated by the instruments great swelling is apt to occur. This is not dangerous but very annoying. A physician can use a conical hand-drill instead of the dental engine, as the bony wall is not thick. Even the stout needle of the aspirator suffices sometimes to force its way into the sinus. If aspiration through a glass tube attached to the canula does not reveal pus the sinus may be irrigated with sterile salt solution. As this flows from the nose into a basin the presence or absence of pus is at once determined.

It has been stated by Ziem, who has had a very large experience, that in some cases pus cannot be found at the time of the puncture, but will issue on the next day. This is not due to a new infection during the operation. I have once had the same experience in a case where pre-existing asthma was at once benefited by operating upon the antrum. It depends probably on the existence of incomplete partitions in the sinus.

While empyema of the antrum may not be attended by any characteristic symptom sufficient for a diagnosis it may yet cause considerable disturbances. These may be neuralgic pains, headaches, nasal stuffiness and discharge and other consequences of nasal irritability, such as asthma, and less commonly derangements of the eyes and ears.

It does not seem to be commonly known that antral disease of nasal origin may heal spontaneously. I have often seen instances of acute coryza in which pus was seen to issue from the nasal orifice of the antrum. And yet after a time all suppuration ceased, nor were there after the recovery any evidences of disease of the antrum. A more positive proof was obtained in a lady in whom I opened the antrum through the canine fossa last September. There was a great deal of pus of mawkish odor but not decomposed. On account of irregular attendance the opening closed after the lapse of a week, at a time when all symptoms, as well as the result of the irrigation two days previously, indicated the continued formation of pus. She then refused another operation. However, as she still has nasal discharge and irritability (and ear disease) I punctured the same antrum again this May, but found no pus on thorough search.

A curious observation which I have made twice was the removal of symptoms after the irrigation of the antrum where the operation did not confirm the diagnosis but revealed absolutely no pus. Once it was discharged from the nose, in the other case ciliary irritation and neuralgia without ocular lesions.

In regard to treatment, I can add but little differing from general experience. In cases dating back no longer than a few months the drainage of the pus is sufficient for a cure. By opening the antrum, irrigating it with an indifferent sterile fluid and blowing in boracic acid we can cure the recent cases in from two to six weeks. A much longer time is required for cases of long standing, sometimes more than one year. In such chronic instances the opening should be made large. The easiest operation is from the the alveolar process, if any teeth have been or are to be extracted or if there be room between them. But as this route does not, as a rule reach the lowest part of the antrum, Kuester's operation through the canine fossa is often more satisfactory. In the latter case a drainage tube can be worn; in the alveolar operation this is superfluous and even an obturator adapted by the dentist to keep the fistula open is of questionable service. One possible reason for the tedious recovery of old chronic cases is the occasional hypertrophy of the mucous membrane lining the antrum amounting even to a polypoid condition. Through a sufficiently large opening, I have used a curette with decided subsequent improvement and with but little pain in two such instances. Antiseptics have seemed to me to be of no greater influence than irrigation with salt water when the pus is copious. As the secretion is diminishing it is doubtful whether irrigation is at all necessary. The pus formation is distinctly checked by blowing into the cavity boracic acid or other soluble powders of similar antiseptic properties. In the more tedious class of cases this can be entrusted to an intelligent patient.

DISCUSSION.

Dr. M. H. Fletcher said that he had had but little experience in cases of this kind, but related the case of one of his patients whom he took to a specialist for operation. The operator attempted to make an opening into the antrum through the canine fossa with a chisel, and found it so difficult that the patient was exhausted before an entrance was effected, and had to return home for rest. Coming back another day to have the operation completed, entrance was effected with a drill. The specialist said there had been a very unusual thickness of the bone; and Dr. Fletcher wished to ask whether such trouble was often found.

Dr. Gradle said that while there were variations in the thickness of the bone, it was rarely that any serious trouble was found.

Dr. E. S. Talbot said he had seen a case similar to the one mentioned by Dr. Fletcher, where the dentist had drilled into the nasal cavity instead of into the antrum. In many cases the antrum does not extend forward to the canine fossa, and if it does it will not extend more than one-fourth to one-half inch back of the third molar. The operator in the case of Dr. Fletcher may have pierced the bone anterior to the antrum into the nasal cavity. Then besides, the antrum is sometimes divided by septa of bone. He believes the better way is to drill into the lower part of the cavity. When this is done it is easy to wash out the cavity with antiseptics, as the drainage will be good and the trouble will be cured.

Dr. Fletcher asked if the peculiarity of the extent or position of the antrum was indicated in any way by the shape of the face. For instance, is the antrum farther back in a face with high cheek-bones, or is there any similar indication in regard to it?

Dr. Talbot said that as far as he knew there was nothing to indicate the extent or position of the antrum from the form or character of the bones of the face.

Dr. J. S. Marshall hoped that something would be said of antral disease following the grip. He had had four cases, in each of which there had been discharges into the nasal cavity of thick mucus—not bad smelling, nor was it pus, but simply apparently an excess of mucus which had filled the cavity and was discharged through the nose. He thought it advisable to open into the antrum between the roots of the second and third molars, as this is usually the most dependent part of the cavity. He usually used, as an antiseptic, Thiersch's solution—a bland antiseptic and very satisfactory:

Boracic acid, 12 parts;
Salicylic acid, 4 parts;
Water, 1000 parts.

Dr. Talbot reported a case where, after the opening had been made, there was no immediate flow of pus; but such a flow did occur a day or two after the operation.

Dr. Gradle, in reply, stated that this had happened in a case of his where he had opened into the antrum, supposing a formation of pus there was responsible for a train of neuralgic troubles. He found no pus at the time of the operation, but in a day or two afterward there was a flow which he was at first inclined to charge to infection from the instrument used in the operation, but the history of the case convinced him that the disease was the cause of the original trouble, as after the antrum was cleaned out the neuralgic troubles ceased. It is possible sometimes that there may be infection by the instrument or if there be morbid fluid already present, piercing the wall and allowing the entrance of air might cause the formation of pus.

Dr. Taft asked Dr. Gradle what per cent. of diseases of the antrum were caused by diseased teeth.

Dr. Gradle thought between forty and fifty per cent.

Dr. Taft raised the question whether the micro-organisms in the pus from such positions as the antrum and the seat of a whitlow, for instance, where there was no opportunity for the microbes to obtain entrance from without, were the cause of the pus-formation, or whether they were merely an accompaniment, and asked where they came from and how they obtained access to such closed-in positions.

Dr. Gradle thought that the blood was possibly the vehicle which carried the microbes.

Dr. A. E. Baldwin said if this was so and the blood carried these micro-organisms, there was certainly no safety in any measures which we could devise to keep them out of the system.

Dr. Clifford thought it better to pay more attention to systemic treatment in diseases of the antrum rather than to depend altogether on local treatment. The cause must in many cases be systemic, and we cannot reasonably expect to effect a permanent cure unless we remove the cause. He had had cases which he relieved without opening into the antrum or establishing any drainage, by paying attention to remedial measures applied to the system at large.

